# Blood Concentrations of Folic Acid and Homocysteine Are Associated with Treatment-Resistant Depression Among Female Depressed Patients

**DOI:** 10.3390/biom16010070

**Published:** 2026-01-01

**Authors:** Iva Radoš, Zrinka Vuksan-Ćusa, Jakov Milić, Marina Šagud, Ana Lončar Vrančić, Nenad Jakšić, Bjanka Vuksan-Ćusa, Nela Pivac

**Affiliations:** 1 Department of Psychiatry and Psychological Medicine, University Hospital Center Zagreb, 10000 Zagreb, Croatia; iva.mahnik@gmail.com (I.R.); marinasagud@mail.com (M.Š.); njaksic@kbc-zagreb.hr (N.J.); bjanka.vuksan@gmail.com (B.V.-Ć.); 2 School of Medicine, Josip Juraj Strossmayer University, 31000 Osijek, Croatia; 3 Vuk Vrhovac University Clinic for Diabetes, Endocrinology and Metabolic Diseases, Merkur University Hospital, 10000 Zagreb, Croatia; zrinka.vuksan@gmail.com; 4 Catholic Faculty of Theology, University of Zagreb, 10000 Zagreb, Croatia; milic.jakov@gmail.com; 5 School of Medicine, University of Zagreb, 10000 Zagreb, Croatia; 6 Department of Laboratory Diagnostics, University Hospital Center Zagreb, 10000 Zagreb, Croatia; ana.loncar.vrancic@kbc-zagreb.hr; 7 Laboratory for Molecular Neuropsychiatry, Division of Molecular Medicine, Rudjer Boskovic Institute, 10000 Zagreb, Croatia; 8 Department of Psychiatric Investigations, University Psychiatric Hospital Vrapce, 10090 Zagreb, Croatia

**Keywords:** cross-sectional design, depressive disorder, treatment-resistant depression, folic acid, vitamin B12, homocysteine

## Abstract

Treatment-resistant depression (TRD) is a subtype of major depressive disorder (MDD) that fails to respond to first-line pharmacotherapy. This cross-sectional study compared blood concentrations of folic acid, vitamin B12, and homocysteine between female depressed patients with or without TRD, and examined the association of these parameters with the severity of depression. It included 116 female patients treated for MDD, of whom 59 (51%) developed TRD. The diagnosis of MDD was established via a structured clinical interview, while the severity of depression was measured with the Montgomery–Asberg Depression Rating Scale. Blood samples were taken at the initial psychiatric examination to determine the serum levels of folic acid and vitamin B12 and plasma levels of homocysteine. Folic acid levels were significantly lower in the female TRD group (*p* < 0.001), whereas homocysteine levels were significantly higher in the female TRD group (*p* < 0.001), compared to the female depressed group without TRD. In the regression analyses, higher levels of homocysteine (*p* < 0.001) were associated with TRD, while lower levels of folic acid (*p* = 0.036) were related to higher severity of depression, independently of sociodemographic and clinical parameters. Our findings showed that folate correlated with symptom severity, while homocysteine correlated with the TRD status in female MDD patients.

## 1. Introduction

Treatment-resistant depression (TRD) is a subtype of major depressive disorder (MDD) that fails to respond to usual first-line pharmacotherapeutic interventions; most frequently, it is defined as an unsuccessful response to at least two different antidepressant medications with appropriate dosages and length of treatment [1]. In fact, the existing literature suggests that around 30% of patients with MDD are resistant to conventional psycho-pharmacotherapy [2]. Moreover, TRD is associated with poorer quality of life, unemployment, large direct and indirect health care costs, prolonged periods of inpatient treatment [1,3], a higher number of lifetime suicidal attempts, and greater intensity of current suicidal ideation [4].

Possible biological pathways implicated in TRD are those related to impaired serotonin and glucocorticoid receptor function, reduced concentrations of plasma coenzyme Q10 and brain-derived neurotrophic factor (BDNF), decreased cerebral spinal fluid (CSF) levels of substance P, and impaired negative feedback system in hypothalamic–pituitary–adrenal (HPA) axis activity [5,6,7]. The moderating role of one-carbon metabolism cycle (including folic acid, vitamin B12, and homocysteine) in antidepressant treatment response was reported [8]. The first step is the conversion of synthetic or dietary folate to 5-methyltetrahydrofolate (L-methylfolate), followed by the conversion of homocysteine into L-methionine via L-methylfolate, while vitamin B12 helps carry out this process [6]. The important role of B vitamins is in DNA methylation and the regeneration of methionine for the folate cycle via clearance of homocysteine [9]. If one nutrient is lacking, this can lead to a defective cycle, or hyperhomocysteinemia, which might increase the risk of depression [10,11], as elevated homocysteine levels are detected in patients with mood disorders [10,11]. Since components of the one-carbon cycle are necessary for the methylation of monoamines, as well as the synthesis and regulation of serotonin and dopamine, they have a clear role in response to antidepressant treatment. Indeed, a relation between folate deficiency and MDD [6], or one-carbon agents and clinical severity of MDD [8] was reported, while individuals with such deficiency might be more prone to TRD [12]. The effectiveness of both folic acid and L-methylfolate, as an adjunctive antidepressant treatment, was found [13,14]. However, additional studies are needed since one meta-analysis reported a lack of conclusive evidence on the antidepressant effects of folic acid and vitamin B12 [15]. Therefore, further investigations that might detect these associations beyond the influence of sociodemographic and clinical parameters commonly linked to the severity of MDD (e.g., age, education level, work status, family history of depression, smoking, and alcohol consumption) [16,17] are warranted.

Due to the inconsistent findings on the role of folic acid, vitamin B12, and homocysteine in depression, particularly with regard to treatment resistance, the aim of this study was to compare blood concentrations of these one-carbon metabolism agents between female patients with TRD and those with depression who responded to treatment (referred to as those without TRD). Furthermore, we also investigated the associations of folic acid, vitamin B12, and homocysteine levels with the severity of depressive symptoms, while controlling for the influence of relevant sociodemographic and clinical parameters. The hypothesis was that TRD patients would have higher homocysteine and lower folic acid and vitamin B12 levels compared to patients without TRD.

## 2. Materials and Methods

### 2.1. Clinical Data for Subjects

This study included 116 adult female patients who were treated for major depression at the Department of Psychiatry and Psychological Medicine, University Hospital Center Zagreb, and the patient recruitment and data collection lasted for 2 years (2021–2022). The patients were diagnosed via a structured clinical interview based on the International Statistical Classification of Diseases and Related Health Problems 10th Revision (ICD-10) diagnostic criteria for depression, and the following ICD-10 diagnoses were among the inclusion criteria: F32, F32.1, F32.2, F32.8, F33, F33.1, F33.2, and F33.4. Thus, all patients had a recurrent depressive disorder. Only a few patients were in remission, and therefore, the analysis was not separately conducted to test for differences between those who were in remission and those who were not. Exclusion criteria in this study were as follows: being older than 65, other major mental disorders (such as, but not limited to, bipolar disorder, schizophrenia and eating disorders), current treatment with antipsychotics and/or mood stabilizers, folic acid and/or vitamin B supplements in the last 6 months, other prescription drugs known to interact with folic acid and vitamin B12 (e.g., corticosteroids, anticonvulsants, gout medication, antacids, proton-pump inhibitors), diagnosis of substance abuse, unregulated endocrine and/or cardiovascular disease, inflammatory bowel disease, celiac disease, autoimmune atrophic gastritis, Imerslund–Grasbeck syndrome, AIDS, pregnancy and lactation, currently on a vegan diet, hemodialysis and peritoneal dialysis, parenteral nutrition, and intellectual disabilities and/or low comprehension skills. After the presence of MDD was confirmed, the diagnosis of TRD was subsequently established according to the general agreement on the definition of TRD [1]. Of the total 116 female patients, 57 (49%) patients were suffering from depression who responded to treatment, whereas 59 (51%) patients had TRD. Treatment response was defined as a ≥50% reduction from baseline in Montgomery–Asberg Depression Rating Scale (MADRS) [18] scores. Consequently, partial responders were considered responders if they had a ≥50% reduction from baseline MADRS scores after at least 8 weeks of treatment. Likewise, they were supposed to have TRD in the case of less than 50% reduction in baseline MADRS total scores. The minimum duration for each antidepressant trial was at least eight weeks, with a dosage equivalent of 40 mg of fluoxetine, which includes 20 mg of Escitalopram, 100 mg of Fluvoxamine 40 mg of Paroxetine, 100 mg of Sertraline, 60 mg of Duloxetine, 150 mg of Venlafaxine, 45 mg of mirtazapine, and 20 mg of vortioxetine. The treatment response was defined in the current episode, at the time of rating, in this cross-sectional study.

Furthermore, blood samples were taken at the initial psychiatric examination in order to determine serum levels of folic acid and vitamin B12 and plasma levels of homocysteine. Each patient received detailed information about this study from their psychiatrist and was asked to sign a formal consent form to participate. Participation was voluntary, and the participants were also informed they could withdraw their consent at any time. The study was approved by the Ethics Committee of the University Hospital Centre Zagreb and carried out in accordance with the Helsinki Declaration.

### 2.2. Blood Sample Procedures and Laboratory Analyses

Blood samples were collected at 7:30 a.m. after an overnight fast during routine laboratory visits. Folic acid and vitamin B12 blood serum concentrations were based on venous samples in a test tube vacuum with a coagulator factor and gel separator (Vacuette, Greiner Bio-One GmbH, Kremsmunster, Austria). Homocysteine blood plasma concentrations were based on venous samples in a test tube vacuum with the anticoagulant K3EDTA (Vacuette, Greiner Bio-One GmbH, Kremsmunster, Austria), and these samples were immediately placed into a mixture of water and ice. Further, folic acid and vitamin B12 concentrations from the serum samples were analyzed in the immunochemical model of Roche Cobas 6000 CEE (Roche Diagnostics International, Rotkreuz, Switzerland). The determination method was electrochemiluminescence. Concentrations of plasma homocysteine were determined by using a tandem mass spectrometer with a high-performance liquid chromatography (HPLC) Shimadzu 8050 (Shimadzu, Kyoto, Japan), and reagent Recipe ClinMass/ClinSpot LC-MS/MS Complete Kits Homocysteine in Plasma/Serum (RECIPE Chemicals + Instruments GmbH, Munich, Germany). All the above-mentioned analyses were carried out at the Department for Laboratory Diagnostics of the University Hospital Center Zagreb, in line with the good laboratory practice. Serum samples were analyzed immediately after they were brought to the Department of Laboratory Diagnostics, while plasma samples were aliquoted into secondary tubes and stored in a −80 °C freezer.

Vitamin B12 test linearity was 36.9 to 1476 pmol/L, while in the case of folic acid, it was between 1.36 and 45.4 nmol/L (based on the blind test limit and the highest master curve calibrator value). Homocysteine levels lower than 5.0 µmol/L were reported as <5.0 µmol/L. If the homocysteine level was higher than the upper limit of the tested linearity, the sample was diluted via HPLC-Liquid Chromatography-Mass Spectrometry Grade (LC-MS Grade) or via a low homocysteine concentration sample until it was an analytically acceptable value. Finally, all the samples used in this study were visually assessed for hemolysis and deemed acceptable for further analyses.

### 2.3. Sociodemographic and Clinical Assessment

Sociodemographic and clinical data were obtained during the initial clinical interview, and the additional information was taken from the hospital chart records. The severity of depressive symptoms was assessed by the Montgomery–Asberg Depression Rating Scale (MADRS) [18]. The Cronbach Alpha coefficient of this rating scale was 0.79. MADRS ratings were performed by trained raters who were blind to biomarker values.

### 2.4. Statistical Analysis

The Kolmogorov–Smirnov test revealed that the most relevant continuous variables used in this study, namely age, disease duration, levels of folic acid, vitamin B12, and homocysteine, as well as depression scores on the MADRS, were non-normally distributed (*p* < 0.05). Therefore, we used the non-parametric Mann–Whitney test to examine the differences in continuous variables between the two clinical groups. The Chi-square test was also used to assess the differences in elevated homocysteine (>15 µmol/L) and folate deficiency (<6.8 nmol/L) between the two clinical groups. Internal reliability of the clinical rating scale (MADRS) was determined through the Cronbach Alpha coefficient. Statistical analyses included methods of descriptive statistics (medians, interquartile ranges, frequencies, and percentages). Differences between the two clinical groups (TRD vs. depression without TRD) in sociodemographic, clinical, and biological parameters were tested with the chi-square and Mann–Whitney tests. Three binary logistic regressions were carried out in predicting the two clinical groups via folic acid, vitamin B12, and homocysteine, while controlling for the influence of possibly relevant sociodemographic and clinical factors. In the case of folic acid, due to the complete separation of the two clinical groups, a Firth-penalized logistic regression analysis was used. Finally, we performed multivariate linear regression analyses with the Enter method in order to examine independent associations of folic acid, vitamin B12, and homocysteine with the severity of depressive symptoms, while controlling for the influence of sociodemographic and clinical variables. Some categorical variables, namely work and relationship status, were transformed into dummy variables. Here, variance inflation factors (VIFs) and residual normality criterion were checked with regard to potential multicollinearity issues and transformation of independent variables, respectively.

G*Power 3 Software was used for the sample size calculation and power analyses. For the Mann–Whitney *t*-test (with *p* < 0.05; power = 0.800; medium effect size = 0.50), the total desired sample size was 102, N = 51 per group, and the actual sample size was 116, with 56 and 59 per group, so the study had an adequate sample size and desired power. All the statistical analyses were carried out in SPSS for Microsoft Windows, version 21.

## 3. Results

### 3.1. Sociodemographic and Clinical Variables in Female Depressed Patients with or Without TRD

First, a series of chi-square tests was performed in order to test the differences in non-continuous sociodemographic and clinical variables between the two clinical groups (Table 1). Patients with depression (without TRD) and those with TRD did not differ significantly (chi-square test) in education, work status, relationship status, family history of mental disorders, alcohol consumption, and smoking status (Table 1).

### 3.2. Clinical and Biological Variables in Female Depressed Patients with or Without TRD

The Mann–Whitney test was performed to examine possible differences between these two clinical groups in age, treatment duration, folic acid, vitamin B12, homocysteine, and depressive symptom severity (MADRS scores). Folic acid levels were significantly lower in the TRD group (*p* < 0.001), whereas homocysteine levels were significantly higher in the TRD group (*p* < 0.001) compared to corresponding values in patients with depression without TRD. Vitamin B12 levels were slightly (*p* = 0.053) lower in TRD compared to the depressive group without TRD, but this difference did not reach the formal level of significance (Table 2). No differences were detected in age, lifetime treatment duration, and MADRS scores between these two groups (Table 2). Furthermore, elevated homocysteine levels (>15 µmol/L) were detected in 9 patients with TRD and in 4 patients without TRD, and this difference was marginally significant (Χ^2^ = 3.998, *p* = 0.046). Folate deficiency (<6.8 nmol/L) was observed in 13 patients with TRD and in none of the patients without TRD, and this difference was significant (Χ^2^ = 14.144, *p* < 0.001).

### 3.3. Prediction of Treatment-Resistant Depression (TRD) in All Female Patients Using Blood Folic Acid, Vitamin B12, and Homocysteine

Binary logistic regression analyses were carried out to predict TRD in all patients (with or without TRD) using folic acid, vitamin B12, and homocysteine, respectively, while controlling for the influence of relevant sociodemographic and clinical factors. Of the three possible predictors, a higher homocysteine level was significantly and uniquely associated with having a diagnosis of TRD (*p* < 0.001), and these findings are shown in Table 3. This binary logistic model had a Negelkerke R^2^ = 0.474. Vitamin B12 was not a significant predictor of TRD (B = 0.001, Exp(B) = 0.999, *p* = 0.330). In the case of blood folic acid, it exhibited complete separation (i.e., perfect discrimination) between patients with and without TRD, preventing reliable estimation of all model parameters in standard logistic regression. This is further evident from the distribution of folic acid levels between patients with and without TRD in Table 2. Thus, a Firth-penalized logistic regression analysis was performed, where higher folic acid concentrations were strongly associated with a reduced likelihood of treatment resistance (B = −2.20, Exp(B) = 0.111, *p* < 0.001).

### 3.4. Prediction of Depression Severity (MADRS) in All Female Patients Using Blood Folic Acid, Vitamin B12, and Homocysteine

In order to assess unique associations of folic acid, vitamin B12, and homocysteine blood levels with the severity of depressive symptoms (using the MADRS scores), a multivariate regression analysis was carried out (Table 4). Here, these associations were tested while simultaneously controlling for the influence of other sociodemographic and clinical parameters. Of all the investigated parameters, lower blood levels of folic acid in female patients were significantly associated with more severe symptoms of depression (β = −0.296, *p* = 0.036). Levels of vitamin B12 (β = 0.126, *p* = 0.215) and homocysteine (β = −0.075, *p* = 0.532) were not significant predictors of depression severity. The overall regression model was significant (F = 2.066, *p* < 0.05), and it explained 12.5% of the variance in the severity of depression (R^2^ = 0.125). Of note, no significant collinearity issues were detected (all the VIFs < 2.3), and no transformation of predictor variables was needed based on the residual normality criterion.

## 4. Discussion

The main results of this cross-sectional study were decreased blood levels of folic acid and increased levels of homocysteine in female patients with depression resistant to treatment, as compared to depressed patients who responded well to treatment. These findings were controlled for the sociodemographic and clinical confounders. Additionally, as far as we are aware, this is the first study to reveal a unique association between lower levels of folic acid and higher intensity of depressive symptoms in the entire sample, which was independent of the effects of other sociodemographic, clinical, and biological parameters. Therefore, our results revealed that folate correlated with symptom severity, while there was a correlation between homocysteine and TRD status in female depressed patients.

These findings are in line with previous, more general, research on these biological markers in MDD: low levels of folate have been found in serum, erythrocytes, and even CFS among depressed patients [8,19,20]. Folate has an effect on the synthesis rate of tetrahydrobiopterin (BH4), a cofactor in the hydroxylation of phenylalanine and tryptophan, making its role vital in the production of monoamine neurotransmitters that are closely related to the development of depression [20]. The relationship between nutrition and depression, however, appears bidirectional. While poor baseline lifestyle habits, which include a lower number of fruit and vegetable servings, are associated with the increased risk of subsequent depression [21], reduced appetite and weight loss are among the crucial symptoms of depression. Therefore, it is unclear if poor nutrition, which may be accompanied by low micronutrient levels including folic acid, may predispose to depression, or depression in turn contributes to the low folate levels, or both. However, the efficacy of antidepressants is also affected by the initial blood folate levels, leading to a less effective drug-based treatment in the case of folate deficiency [12,14]. These patients might perhaps be labeled as ‘’treatment-resistant’’, but more research, like our current study, is needed to uncover the specific influence of folate deficiency in the development of TRD [6]. Folic acid emerged as a strong and statistically robust predictor of TRD, even after applying Firth-penalized logistic regression to account for complete separation in the biomarker values. Although the direction of this association is biologically plausible and consistent with the above-mentioned literature, the magnitude of the effect should be interpreted cautiously due to the limited overlap in folic acid concentrations between groups. Due to the possibility that this effect reflects sampling characteristics rather than a stable biological marker, replication in larger and more heterogeneous cohorts is necessary. In fact, studies have demonstrated the effectiveness of folic acid and L-methylfolate as both a monotherapy and as an adjunctive therapy in MDD [13,14,22], but recent longitudinal studies and systematic reviews are still inconclusive, although promising with regard to its safety and long-term management of specific MDD subpopulations [15,23,24]. Similarly, we also found higher blood levels of homocysteine in the TRD group compared to non-treatment-resistant depressed patients, as well as a unique link between higher homocysteine levels and TRD, independent of sociodemographic and clinical factors. This is partly in accordance with previous research on the role of hyperhomocysteinemia in MDD [11,19], while more studies are needed to assess the unique effects of homocysteine on the antidepressant-based treatment of MDD and, consequently, the development of TRD. Given the complex interplay between folic acid and homocysteine in the synthesis of neurotransmitters that are involved in the development of depression, such as serotonin and dopamine, both these one-carbon agents could possibly be considered as biological markers of TRD [6,20]. Indeed, some authors have pointed out that co-treatment with antidepressants and folic acid results in an improved efficiency that correlates with changes in homocysteine levels [25], and that this co-treatment may alter lymphocyte function in depressed patients indirectly by reducing homocysteine levels [22]. In our study, we did not detect significantly decreased vitamin B12 blood levels in TRD compared to treatment-nonresistant patients (namely, vitamin B12 reduction did not reach the level of statistical significance in TRD), a novel finding in this field that warrants replication in future studies. Although one longitudinal study showed an increased likelihood of developing depressive symptoms over 4 years in older adults with low B12 status [26], the role of vitamin B12 in the treatment of MDD, particularly in its resistant sub-form and in non-geriatric populations, is still under debate [15,27].

Our results revealed a unique association between a lower level of folic acid and more severe symptoms of depression, beyond the influence of relevant sociodemographic, clinical, and biological parameters, including vitamin B12 and homocysteine. This was demonstrated in the total sample of MDD patients, further corroborating the role of folic acid, not just in the treatment resistance, but also in the clinical picture of depression in terms of its intensity. Partly in line with this finding, lower levels of folic acid may contribute to depression and affect its duration and degree of clinical severity [20]. In our analysis, more active smoking was also associated with a higher level of depressive symptoms, a finding in agreement with previous data [28]; however, our results revealed no significant differences in smoking status between patients with or without TRD. The complex causal pathway between smoking and depression is still not fully understood [29], but smoking is undoubtedly associated with comorbid somatic diseases in persons with MDD [30]. In addition, smoking may reduce serum levels of several antidepressants, such as fluvoxamine, duloxetine, mirtazapine, and trazodone [31], which may compromise their efficacy. Of note, the overall regression model explained only 12.5% of the variance in depression severity, confirming that MDD is a complex heterogeneous syndrome influenced by numerous and interrelated biological (including genetic), early-life, societal, life-style, and cultural determinants [16,17].

The relationship between folic acid and poor response to antidepressants is not completely understood. L-methylfolate, a metabolically active form of folic acid, appears to facilitate monoamine synthesis. For example, in a preclinical model of folate deficiency, reduced levels of dopamine and serotonin were observed, along with increased markers of inflammation, such as increased circulatory interleukin 6 (IL-6) and CRP levels [32]. On the contrary, in a rat model of depression, adding folic acid decreased serum corticosterone levels, increased BDNF expression in the hippocampus and in the associative cortex, and the number of spine synapses [33]. In mice with an alcohol-induced brain injury, treatment with folic acid reduced neuroinflammation and enhanced mitochondrial function [34]. Given the abundant evidence on the altered monoamine neurotransmission, elevated inflammatory parameters, and decreased markers of neuroplasticity, such as BDNF in major depression [35], these findings collectively suggest that folate deficiency may contribute to both the onset of depression and poor response to antidepressants.

High levels of homocysteine were associated with the overstimulation of N-methyl-D-aspartate (NMDA) receptors in the hippocampus, which resulted in sustained calcium influx, mitochondrial reactive oxygen species generation, and neurotoxicity [36]. In post-stroke rats, homocysteine treatment decreased synaptic density in the hippocampus and lowered brain monoamine levels [37]. Therefore, increased homocysteine concentration may lead to treatment resistance by interfering with NMDA function and contributing to hippocampal atrophy, which is a well-known finding in patients with MDD [38], particularly in those with treatment resistance [39].

Low intake of several nutrients, including folic acid, was detected in patients diagnosed with depression compared to healthy controls, although this study did not account for treatment resistance and was limited by a small sample size [40]. Such unhealthy eating habits may lead to obesity. Obesity may potentiate cytokine secretion and promote an inflammatory state, which can both worsen the severity of depressive symptoms and further decrease folate levels [41]. However, the link between poor dietary habits and depression appears more complex, given that fast food consumption was connected with depression independent of obesity [42]. Reduced dietary folic acid intake may result in heightened homocysteine levels, which further facilitate inflammation [43]. It remains to be explored whether poor nutrition is more prominent in patients with TRD, and, importantly, if improving the quality of dietary intake may help alleviate the symptoms.

Depression is twice as common in women as in men [44], and women are more likely to experience residual symptoms and treatment resistance [45]. Therefore, in this study, we have included only female patients with depression, with or without TRD. In addition, sex-specific differences in homocysteine [46] and folate [47] levels were detected. Folate levels were lower in males compared to females, which may contribute to the higher homocysteine levels found in males [46,48]. The independent factors associated with increased homocysteine concentration differed between males and females [46]. Namely, lower folate was associated with symptoms of depression in women [47], while higher homocysteine was more linked with depression in middle-aged men [49]. These findings suggest diverse biochemical pathways, influenced by hormones and diet, where folate deficiency impairs neurotransmitter synthesis, potentially increasing depression risk, especially in women [47]. The increased homocysteine levels contribute to the onset of depression and other first-episode psychiatric disorders [49].

In addition to these biological markers related to components of the one-carbon cycle in TRD, glymphatic dysfunction was proposed to be a new neurobiological framework relevant to depression [50,51,52]. Namely, recent research regarding new paradigms of TRD points to a complementary framework involving the glymphatic system, a specialized glial lymphatic pathway that removes metabolic waste products, particularly during deep sleep. Preliminary evidence suggests that enhancing glymphatic function by improving sleep architecture, supporting astrocyte health, affecting the sleep–circadian−mediated pathway and vascular–metabolic–immune pathways, or scheduling drug delivery based on circadian fluctuations, may offer clinical benefits [50]. In addition, although there was no direct proof, recently it has been reviewed that folate might influence glymphatic circulation, and could improve glymphatic clearance, consequently increasing the removal of the neurotoxic waste products in the brain and accordingly might show neuroprotective effects [53]. This speculative hypothesis needs to be confirmed in future research, and might have a wide impact on neuropsychiatric disorders [53]. However, since low levels of folate and higher levels of homocysteine might result in increased oxidative stress, pro-inflammatory cytokines, and neuronal apoptosis, and are associated with cognitive decline and dementia [53], our data on the reduced folic acid and increased homocysteine levels in female depressed patients with TRD suggest that routine cognitive and blood screening is recommended for these patients.

Some limitations of the current research study must be acknowledged. This study was cross-sectional in terms of MDD diagnosis, which prevents us from reaching conclusions about the causal relations between the levels of one-carbon agents and the development of treatment resistance. Since the definition of TRD [1] does not consider non-compliance and poor tolerability (frequently associated with the lack of treatment adherence), and the antidepressant drug levels were not collected, the pseudo-resistance in the TRD group due to poor or non-compliance with treatment cannot be excluded. Furthermore, current pharmacotherapy (type and dose of antidepressant at blood sampling) was not included in the models and is therefore an additional limitation. In addition, eating habits were not measured, whereby natural food folate intake influenced folate levels in the blood. We did not have data on anthropometric measures, such as BMI and abdominal obesity, which are known to be linked to folate and homocysteine metabolism [54]. Therefore, the absence of BMI, dietary intake, metabolic parameters, and inflammatory markers substantially limits interpretability, given their known influence on folate and homocysteine metabolism. Residual confounding, thus, may partially explain the observed associations. Because the design is cross-sectional, it was impossible to determine whether low folate and high homocysteine contributed to TRD or reflected nutritional, metabolic, or illness-related changes in already-resistant patients. Future studies should include the *MTHFR* gene while investigating the role of folate and homocysteine abnormalities in depression, because some *MTHFR* genetic polymorphisms seem to inhibit the conversion of folate into L-methylfolate, possibly making these individuals more prone to developing MDD or even TRD [11]. In addition, there are several definitions of TRD based on different clinical criteria, but in this study, the most broadly, albeit not universally, accepted definition of TRD was used [1]. Also, only female patients were recruited because of the higher prevalence of MDD in women [16] and gender-specific pharmacological profiles related to TRD [55], so our findings cannot be generalized to male patients with TRD, and therefore, novel studies should include male patients with TRD. Finally, the inclusion of multiple predictors in regression analyses raises the possibility of Type I error inflation. However, the variables were selected a priori based on established clinical and biological relevance, and were evaluated within a single multivariable regression model. No formal correction for multiple testing was applied, as such adjustments may be overly conservative in multivariable contexts and increase Type II error risk. Nevertheless, results should be interpreted with caution, and replication in larger samples is warranted.

Strengths of this study are in the evaluation of subsequent development of TRD and comparison between the two depressive groups of female patients in numerous sociodemographic, lifestyle, and clinical parameters (e.g., age, education level, work status, relationship status, family history of psychiatric disorders, alcohol consumption, smoking status, treatment duration, and depressive symptom severity), the rigorous psychiatric evaluations performed by psychiatrists and clinical psychologist, the inclusion of the same ethnic groups, and in the control of the biological variables for possible confounders.

## 5. Conclusions

In this cross-sectional study, lower folic acid and, in particular, higher homocysteine levels were detected in female depressed patients with TRD compared to female depressed patients who responded well to treatment. These findings cannot be generalized beyond female patients with MDD. Therefore, folate was correlated with symptom severity, while homocysteine was correlated with the TRD status in female MDD patients, suggesting that routine blood assessment should be promoted in female MDD patients, although further studies with larger samples and male subjects are still warranted.

## Figures and Tables

**Table 1:** Differences in non-continuous sociodemographic and clinical parameters between female depressed patients with and without treatment-resistant depression (TRD).

Parameter		Patients Without TRD (*n* = 57)	Patients with TRD (*n* = 59)	Χ^2^; *p*
Education	Primary school	8 (14%)	10 (17%)	0.460; 0.794
	Secondary school	41 (72%)	39 (66%)	
	Higher education	8 (14%)	10 (17%)	
Work status	Employed	11 (19%)	13 (22%)	0.319; 0.853
	Unemployed	28 (49%)	30 (51%)	
	Retired	18 (32%)	16 (27%)	
Relationship status	Not married	3 (5%)	5 (8%)	4.747; 0.191
	Married	43 (75%)	39 (66%)	
	Divorced	4 (7%)	11 (19%)	
	Widowed	7 (13%)	4 (7%)	
Family history of mental disorders	No	41 (72%)	45 (76%)	0.285; 0.593
	Yes	16 (28%)	14 (24%)	
Alcohol consumption	No	53 (93%)	58 (98%)	1.991; 0.158
	Yes	4 (7%)	1 (2%)	
Smoking status	No	37 (65%)	39 (66%)	0.018; 0.893
	Yes	20 (35%)	20 (34%)	

Χ^2^ —the chi-square test; *p*—statistical significance of the chi-square test; TRD—treatment-resistant depression.

**Table 2:** Differences in continuous sociodemographic, clinical, and biological parameters between female depressed patients with and without treatment-resistant depression (TRD).

Parameter	Patients Without TRD (*n* = 57)	Patients with TRD (*n* = 59)	Z; *p*	r
Age (years)	56 (20–61)	55 (30–65)	−0.592; 0.554	−0.055
Folic acid nmol/L	17.6 (12.6–45)	8.8 (4.5–12)	−9.287; 0.000	−0.862
Vit B12 pmol/L	292 (141–677)	261 (115–862)	−1.933; 0.053	−0.179
Homocysteine µmol/L	5 (5–19)	12 (6–41)	5.297; 0.000	0.492
MADRS scores	32 (7–50)	34 (22–46)	1.062; 0.288	0.099
Lifetime treatment duration (years)	8 (1–27)	7 (1–28)	−0.581; 0.561	−0.054

Z—standardized test statistic of the Mann–Whitney test; *p*—statistical significance of the Mann–Whitney test; r—standardized effect size of the Mann–Whitney test; MADRS—Montgomery–Asberg Depression Rating Scale; TRD—treatment-resistant depression.

**Table 3:** Binary logistic regression predicting treatment-resistant depression (TRD) using blood homocysteine levels in total sample of 116 female patients with depression.

Predictor	B (S.E.)	Wald	Exp(B) [95% CI]	*p*
Age	−0.057 (0.029)	3.792	0.945 [0.892, 1.000]	0.053
Education level	0.397 (0.516)	0.594	1.488 [0.542, 4.087]	0.441
Number of children	0.136 (0.259)	0.275	1.146 [0.689, 1.905]	0.600
Smoking status	0.147 (0.588)	0.063	1.159 [0.366, 3.671]	0.803
Alcohol consumption	−3.333 (1.731)	3.707	0.036 [0.001, 1.062]	0.055
Family history of mental disorders	−0.185 (0.601)	0.095	0.831 [0.256, 2.700]	0.758
Homocysteine µmol/L	0.291 (0.064)	20.546	1.338 [1.180, 1.518]	0.000

*p*—statistical significance of each predictor in the model; B—non-standardized coefficient of predictor variables; S.E.—standard error; Wald—Wald test; Exp(B)—prognostic value for each predictor; CI—confidence interval for Exp(B).

**Table 4:** Multivariate regression analysis predicting depression severity (MADRS) using blood folic acid, vitamin B12, and homocysteine in total sample of 116 female patients with depression.

Predictor	B [95% CI]	β	*p*	F	R^2^
Age	−0.050 [−0.186, 0.085]	−0.079	0.495	2.066 *	0.125
Folic acid nmol/L	−0.213 [−0.404, −0.022]	−0.296	0.036		
Vit B12 pmol/L	0.005 [−0.003, −0.014]	0.126	0.215		
Homocysteine µmol/L	−0.068 [−0.265, 0.128]	−0.075	0.532		
Relationship status	−1.287 [−3.016, 0.442]	−0.133	0.224		
Education level	−1.274 [−3.430, 0.881]	−0.103	0.327		
Number of children	0.700 [−0.531, 1.932]	0.119	0.245		
Work status	0.243 [−1.671, 2.157]	0.011	0.923		
Family history of mental disorders	1.671 [−0.944, 4.286]	0.142	0.161		
Alcohol consumption	−3.244 [−8.993, 2.505]	−0.120	0.261		
Smoking status	4.534 [1.882, 7.186]	0.488	0.007		
Clinical group (TRD vs. patients without TRD)	−1.073 [−4.429, 2.283]	−0.084	0.562		

MADRS—Montgomery–Asberg Depression Rating Scale; B—non-standardized regression coefficient; CI—confidence interval for B; β—standardized beta coefficient; *p*—statistical significance of each predictor in the model; F—overall significance of the regression model; R^2^—coefficient of determination (amount of variance explained); TRD—treatment-resistant depression; *—significant.

## Data Availability

The datasets used and/or analyzed during the current study are available from the corresponding author on reasonable request.

## Abbreviations

The following abbreviations are used in this manuscript:

AIDS	Acquired immunodeficiency syndrome
BDNF	Brain-derived neurotrophic factor
CSF	Cerebrospinal fluid
ICD-10	International Statistical Classification of Diseases and Related Health Problems, 10th Revision
HPA	Hypothalamic–pituitary–adrenal
MADRS	Montgomery–Asberg Depression Rating Scale
MDD	Major depressive disorder
TRD	Treatment-resistant depression
